# Risk factors for urinary tract infection in elderly patients with type 2 diabetes: A protocol for systematic review and meta-analysis

**DOI:** 10.1371/journal.pone.0310903

**Published:** 2024-09-26

**Authors:** Hairong Jia, Wenhao Su, Jiaqi Zhang, Zhaoyang Wei, Pepertual Tsikwa, Yanru Wang

**Affiliations:** School of Nursing, Zhejiang Chinese Medical University, Hangzhou, Zhejiang, China; University of Ilorin, NIGERIA

## Abstract

**Introduction:**

Type 2 diabetes mellitus (T2DM) is a frequent chronic condition among the elderly, which increasing their susceptibility to infection. Urinary tract infection (UTI) is one of the most prevalent infections among older people with T2DM. However, the association between geriatric T2DM and the risk of UTI has not been thoroughly researched and is still contentious. Consequently, this protocol describes a systematic review to pinpoint the primary risk factors for UTI among elderly T2DM. Our goal is to improve recommendations for the creation of targeted treatment interventions by examining risk factors for UTI in elderly individuals with T2DM.

**Methods and analysis:**

We will search 4 English literature databases (PubMed, Embase, Web of Science, and Cochrane Library) and 3 major Chinese databases (CNKI, WanFang, and VIP) from the establishment of the database to June 20, 2024. Systematic evaluation and meta-analysis will be conducted on cohort and case-control studies exploring the occurrence and risk determinants of UTI in individuals diagnosed with T2DM. The main focus will be on identifying the risk factors for UTI in elderly diabetic patients. Two researchers will independently review articles, collect data, and evaluate the quality and potential bias of study inclusion. We will use RevMan V.5.4 software to analyze the data. The quality of the included studies will be assessed using the Newcastle-Ottawa scale. In addition, the GRADE (Grade of Recommendations, Assessment, Development, Evaluation) method will be used to examine the quality of evidence for each exposure and outcome of interest.

**Discussion:**

This study aims to illuminate the various risk factors associated with UTI in older patients diagnosed with T2DM. By this thorough investigation, we hope to provide a more comprehensive reference for medical professionals and researchers, thereby supporting the implementation of effective preventive strategies against UTI and improving overall nursing outcomes for this specific patient population.

**Trail registration:**

PROSPERO (CRD42024559129).

## Introduction

Diabetes is a chronic disease characterized by an abnormal metabolism of blood glucose, leading to a significant global health burden [1]. According to an estimate by the International Diabetes Federation, approximately 463 million individuals worldwide have been diagnosed with diabetes in 2019. It is projected that by 2045, the global number of people with diabetes will reach 780 million, with a prevalence rate of 12.2% [2]. The elderly population (≥60 years old) with diabetes is rapidly increasing due to aging and has become the predominant group affected by diabetes [3], with T2DM accounting for over 90% [4].

Previous studies have found that patients with diabetes will have a variety of complications, such as macrovascular complications (including coronary heart disease, stroke, and peripheral vascular disease), microvascular complications (including end-stage renal disease, retinopathy, and neuropathy), dementia, depression, and urinary tract infection (UTI) [5]. Existing evidence shows that compared with non-diabetic patients, people with diabetes are more likely to get specific infections, such as pyelonephritis, asymptomatic bacteriuria, UTI, lower limb infection, and deep subcutaneous tissue infection [6].

UTI is a common infectious disease caused by bacteria or fungi [7]. According to studies, the elderly (65 years of age and older) are more likely to have UTI [8]. UTI is prevalent among senior diabetic individuals. Previous research has shown that roughly 43% of T2DM patients aged 60 and over have bacteriuria [9, 10]. A systematic review and meta-analysis revealed that the prevalence of UTI in patients with T2DM was 11.5%, and the incidence increased with age [11].

Elderly adults with T2DM and UTI may have a subtle beginning and are difficult to identify [12]. If a UTI is not treated promptly, it can progress to severe complications such as emphysematous pyelonephritis or cystitis, perinephric abscess, and renal papillary necrosis [13]. These difficulties not only extend the patient’s hospital stay and raise the expense of therapy [14], but they also have a negative impact on the patient’s quality of life and prognosis [15]. Furthermore, senior diabetic patients are five times more likely to die from a UTI than healthy people [16]. As a result, we need to devise appropriate management measures to prevent UTI in elderly diabetic patients.

There has been little research on UTI in older individuals with T2DM, and the risk variables indicated in various studies are not entirely consistent [17, 18]. Therefore, the purpose of this study is to identify the risks that contribute to the risk of UTI in senior patients with T2DM using a meta-analysis, allowing medical personnel to promptly identify high-risk groups and conduct effective intervention strategies.

## Methods

### Study registration

The initiation of our research is planned for the 20th of June, 2024, and it is anticipated to finish by the end of December, 2024. We will adhere to the PRISMA-P Checklist guideline (S1 File) [19] and conduct a systematic assessment following the Cochrane Handbook. The protocol has been registered with PROSPERO on June 27, 2024, under the registration number CRD42024559129 (S2 File).

## Literature selection

### Inclusion criteria

#### Population

Elderly type 2 diabetes who present with UTI were the main focus of this systematic review and meta-analysis. “Older people” are defined by the UN and WHO as those who are 60 years of age or older. The screening procedure for this study will employ the aforementioned inclusion criterion (age≥60 years), and the results will be categorized by age group.

#### Exposure

Elderly patients with type 2 diabetes mellitus who present with urinary tract infection.

#### Types of studies

This study will include case-control studies or cohort studies.

### Exclusion criteria

- Repeated publications, conference papers, meta-analyses, reviews, protocols, animal studies, and letters;
- Unable to obtain the full text of the literature or incomplete literature data present;
- Low quality literature.The Newcastle-Ottawa Quality Assessment instrument (NOS)<4 indicates low methodological quality evaluations in the literature.

## Search strategy

The following databases will be searched: PubMed, Web of Science, the Cochrane Library, EMBASE, CNKI, WanFang and VIP. Additionally, we will also search grey literature to avoid missing relevant studies. This study will use a combination of medical subject headings (MeSH) and corresponding free terms to search, covering the period from the establishment of the database up until June 2024. Further details on the search approach can be found in the Additional Material (S3 File). The medical subject headings utilized included diabetes mellitus type 2 (T2DM), urinary tract infection (UTI) and risk factors.

## Data collection and analysis

After importing all the acquired literature into the Endnote X9 software, two researchers (J.H.R. and S.W.H.) will independently assess the studies obtained. Using Endnote X9 software, remove duplicated research and review titles along with abstracts to exclude studies not meeting the inclusion criteria. Subsequently, they will thoroughly scrutinize the complete content of the remaining papers to filter out irrelevant research. In the data gathering and evaluation stage, in cases where the two researchers failed to agree, disputes were settled by consulting a third researcher (W.Y.R.). The process for selecting studies is illustrated in S1 Fig.

## Data extraction

The data for this review will be extracted independently by two researchers (J.H.R. and S.W.H.) using pre-established forms. This form will collect the following information: patient-related data (including age, gender, length of hospitalization, disease duration, laboratory findings, complications, the presence of indwelling catheters, and other factors relevant to urinary tract infections), as well as study characteristics (such as the primary author, country, title, publication year, and sample size). In cases where there is missing information, efforts will be made to contact the corresponding author of the study. If no response is received, the paper will be included in the review but excluded from the quantitative analysis.

## Assessment of risk of bias

The evaluation of bias in the study will be conducted separately by two trained researchers (J.H.R. and S.W.H.). In the case of any disagreement, a third investigator (W.Y.R.) will be consulted to reach a final decision. The evaluation of the quality of cohort and case-control studies in this investigation will be conducted using the Newcastle-Ottawa Scale (NOS) [20]. The NOS is structured around three key domains: the selection of participants, the comparability of groups, and the assessment of the exposure or outcome. The scale is divided into eight criteria. It assigns a maximum score of 9, with ratings from 0 to 3 suggesting poor quality, 4 to 6 indicating fair quality, and 7 or above reflecting high quality. This study will only include literature of high and medium quality, while low-quality literature will be excluded.

## Strategy for data synthesis

In our study, RevMan V.5.4 software will be used to conduct a meta-analysis of risk factors from the collected literature. Sensitivity analysis will be performed using Stata 17 software and the one-by-one elimination approach. Dichotomous data will be analyzed using the standard mean difference (SMD) or weighted mean difference (WMD) with a 95% confidence interval (CI). Besides, binary variables will be assessed using the odds ratio (OR), which is accompanied by a 95% confidence interval.

## Assessment of heterogeneity

In this study, the Cochrane’s Q test and the *I*^2^ index will be utilized to assess the heterogeneity among studies. When *p* > 0.1 and *I*^2^ < 50%, the heterogeneity among studies is deemed acceptable, and an analysis is conducted using a fixed effect model. Conversely, if *p* ≤ 0.1 or *I*^2^ ≥ 50%, the heterogeneity among studies is considered significant, leading to the utilization of a random effect model for analysis [21]. If the heterogeneity>75%, then no meta-analysis was performed. We will use a descriptive analysis.

## Quality of evidence and publication biases assessment

The two researchers (J.H.R. and S.W.H.) will evaluate the quality of the evidence using the Grading of Recommendations Assessment, Development, and Evaluation (GRADE) [22]. It aims to assess the quality of the evidence in the integrated findings. The quality of evidence is notably influenced by the following five key factors: the indirectness of the evidence, inconsistencies in the outcomes, limitations within the findings, inaccuracies in the results, and the presence of potential publication bias. In cases where the number of included studies exceeds 10, the publishing bias will be evaluated using funnel plots. Asymmetry on both sides of the funnel plot will suggest that Egger’s experiment and publication bias are more likely to be used. It demonstrates the presence of publication bias if the *p* value (<0.05). Furthermore, it is the reverse funnel plot outcome if *p* > 0.05, at which point we can use the cut-and-patch method to make additional determinations.

## Sensitivity analysis

If there is apparent heterogeneity, specifically when it exceeds 50%, we will perform a sensitivity analysis to evaluate the data’s reliability. The sensitivity analysis method involves eliminating papers one at a time and comparing the statistical analysis results to the original estimates, aiming to evaluate the specific influence of each literature on the final summary findings.

## Ethics and dissemination

There will be no need for patient informed consent or permission from the ethics committee. The systematic review and meta-analysis findings will be published in a peer-reviewed publication.

## Discussion

Many researches have examined the occurrence of UTI in diabetes patients; however, no systematic review has yet adequately evaluated the primary risk factors for UTI in older people with T2DM. Thus, the purpose of this study is to use systematic review and meta-analysis to gain a deeper understanding of the main factors that raise the risk of UTI in older patients with T2DM.

First of all, diabetes is a chronic disease; long-term high blood sugar in the body environment is conducive to bacterial growth and reproduction [23], and the urinary tract itself has a variety of bacterial colonization [24]. Secondly, the autonomic nerve injury caused by long-term hyperglycemia leads to a neurogenic bladder, and urine retention in the bladder makes it easy to breed bacteria and cause UTI [25]. Finally, most of the elderly patients with T2DM are long-term patients; bladder dysfunction and immune function damage are more serious, and the risk of UTI is higher [9].

UTI stands as the prevailing bacterial disease among the elderly demographic [26]. Advancing age brings about a substantial decline in both the physiological and immune functions of elderly individuals with diabetes, significantly diminishing their ability to combat pathogens [27]. Furthermore, the mucosal epithelium of the urinary and reproductive tracts in the elderly undergoes degeneration, further elevating the susceptibility to UTI [28]. Simultaneously, the presence of infection hampers blood sugar regulation, creating a detrimental cycle that perpetuates the deterioration of diabetic patients’ conditions, culminating in chronic ailments accompanied by multiple complications such as diabetic nephropathy, cerebrovascular disease, diabetic retinopathy, diabetic macroangiopathy, and diabetic neuropathy [9]. These complications complicate the clinical management of older individuals with T2DM and may increase patients’ indirect mortality [28].

A considerable number of studies have explored various risk factors associated with UTI in individuals diagnosed with diabetes. These factors encompass age [29], sex [30], medications (primarily SGLT2 inhibitors) [18, 31], coexisting ailments (such as UTI, diabetic nephropathy, and a history of hypertension) [17, 32], and the severity of the condition. It is worth noting that the influence of certain factors, including hospitalization duration, body mass index, and glycemic control, on UTI may vary among individuals with diabetes [11]. Nonetheless, upon careful examination of the available data, we have identified certain limitations in the earlier study. For example, most investigations into risk factors for UTI in diabetes have included patients from different age groups. There is a dearth of specific evidence examining risk factors for UTI in older adults with diabetes. However the “Guidelines for the Treatment of Diabetes in the Elderly (2024 Edition)” in China emphasize significant differences among elderly patients with diabetes [33]. The majority of older individuals with diabetes exhibit compromised immunity [34], overall health conditions, and an increased number of risk factors for urinary tract infections when compared to individuals with diabetes under the age of 60 [27]. Hence, it is imperative to gather data concerning UTI among elderly individuals with a diagnosis of T2DM.

This study will use a rigorous search strategy, including medical subject headings and their corresponding free words and key phrases, to ensure a comprehensive search of the literature. Furthermore, we will conduct rigorous screening, quality assessment, and bias assessment of the literature. However, this study may have some potential limitations. Firstly, if there is excessive heterogeneity among the included studies, conducting a meta-analysis may become impracticable. Secondly, there aren’t many studies that particularly focus on UTI in elderly patients with T2DM because the majority of the literature examines diabetics of all ages who developed UTI. Therefore, our conclusions about risk variables may be impacted if fewer studies are included or if the sample size is insufficient.

## Supporting information

S1 Fig PRISMA flow diagram describing the selection process of studies. (TIF)

S1 File PRISMA-P checklist (2015). (PDF)

S2 File PROSPERO registration document. (PDF)

S3 File The detailed search strategies for databases. (DOCX)
