# Supplementary figures and images for: Risk factors for urinary tract infection in elderly patients with type 2 diabetes: A protocol for systematic review and meta-analysis
Source: PLoS One. 2024 Sep 26;19(9):e0310903. doi: 10.1371/journal.pone.0310903 (PMC11426445; doi:10.1371/journal.pone.0310903)

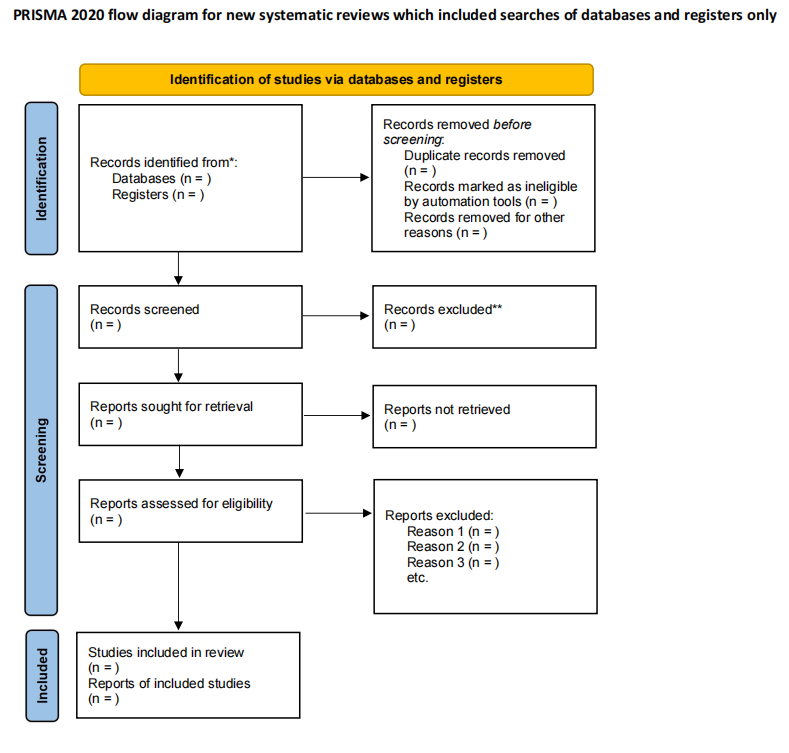

Supplement: S1 Fig — (TIF) [file pone.0310903.s001.tif]
