# Supplementary material for: Risk factors for urinary tract infection in elderly patients with type 2 diabetes: A protocol for systematic review and meta-analysis
Source: PLoS One. 2024 Sep 26;19(9):e0310903. doi: 10.1371/journal.pone.0310903 (PMC11426445; doi:10.1371/journal.pone.0310903)
Supplement: S2 File — (PDF) [file pone.0310903.s003.pdf]

Systematic review

A list of fields that can be edited in an update can be found here

1. * Review title.

Give the title of the review in English

Risk Factors for Urinary Tract Infection in Elderly Patients with Type 2 Diabetes: a protocol for systematic review and meta-analysis

2. Original language title.

For reviews in languages other than English, give the title in the original language. This will be displayed with the English language title.

Risk Factors for Urinary Tract Infection in Elderly Patients with Type 2 Diabetes: a protocol for systematic review and meta-analysis

3. * Anticipated or actual start date.

Give the date the systematic review started or is expected to start.

18/06/2024

4. * Anticipated completion date.

Give the date by which the review is expected to be completed.

31/12/2024

5. * Stage of review at time of this submission.

This field uses answers to initial screening questions. It cannot be edited until after registration.

Tick the boxes to show which review tasks have been started and which have been completed.

Update this field each time any amendments are made to a published record.

The review has not yet started: Yes

Preliminary searches	No	No
Piloting of the study selection process	No	No
Formal screening of search results against eligibility criteria	No	No
Data extraction	No	No
Risk of bias (quality) assessment	No	No
Data analysis	No	No

Provide any other relevant information about the stage of the review here.

6. * Named contact.

The named contact is the guarantor for the accuracy of the information in the register record. This may be any member of the review team.

Hairong Jia

Email salutation (e.g. "Dr Smith" or "Joanne") for correspondence:

Miss Jia

7. * Named contact email.

Give the electronic email address of the named contact.

jhr6771@163.com

8. Named contact address

Give the full institutional/organisational postal address for the named contact.

Department of Nursing, Zhejiang Chinese Medical University, Zhejiang, Hangzhou, China

9. Named contact phone number.

Give the telephone number for the named contact, including international dialling code.

13645416771

10. * Organisational affiliation of the review.

Full title of the organisational affiliations for this review and website address if available. This field may be completed as 'None' if the review is not affiliated to any organisation.

Department of Nursing, Zhejiang Chinese Medical University, Zhejiang, Hangzhou, China

Organisation web address:

11. * Review team members and their organisational affiliations.

Give the personal details and the organisational affiliations of each member of the review team. Affiliation refers to groups or organisations to which review team members belong.
NOTE: email and country now MUST be entered for each person, unless you are amending a published record.
PLEASE USE AN INSTITUTIONAL EMAIL ADDRESS IF POSSIBLE.

Jia HaiRong. Department of Nursing, Zhejiang Chinese Medical University
Mrs Wang Ranru. Department of Nursing, Zhejiang Chinese Medical University
Mr Su Wenhao. Department of Nursing, Zhejiang Chinese Medical University
Zhang Jiaqi. Department of Nursing, Zhejiang Chinese Medical University
Mr Wei Zhaoyang. Department of Nursing, Zhejiang Chinese Medical University

12. * Funding sources/sponsors.

Details of the individuals, organizations, groups, companies or other legal entities who have funded or sponsored the review.

None

Grant number(s)

State the funder, grant or award number and the date of award

13. * Conflicts of interest.

List actual or perceived conflicts of interest (financial or academic).

None

14. Collaborators.

Give the name and affiliation of any individuals or organisations who are working on the review but who are not listed as review team members. **NOTE: email and country must be completed for each person, unless you are amending a published record.**

15. * Review question.

State the review question(s) clearly and precisely. It may be appropriate to break very broad questions down into a series of related more specific questions. Questions may be framed or refined using PI(E)COS or similar where relevant.

Finding risk factors for urinary tract infections in elderly type 2 diabetic patients using meta-analysis and systematic review

16. * Searches.

State the sources that will be searched (e.g. Medline). Give the search dates, and any restrictions (e.g. language or publication date). Do NOT enter the full search strategy (it may be provided as a link or attachment below.)

We will search the following seven databases: CNKI, Wanfang, VIP, PubMed, Embase, Cochrane Library, and Web of Science. The search date ranges from the database's inception to June 18, 2024, without any language or geographical.

17. URL to search strategy.

Upload a file with your search strategy, or an example of a search strategy for a specific database, (including the keywords) in pdf or word format. In doing so you are consenting to the file being made publicly accessible. Or provide a URL or link to the strategy. Do NOT provide links to your search **results**.

Alternatively, upload your search strategy to CRD in pdf format. Please note that by doing so you are consenting to the file being made publicly accessible.

Do not make this file publicly available until the review is complete

18. * Condition or domain being studied.

Give a short description of the disease, condition or healthcare domain being studied in your systematic review.

As the population ages, the number of senior individuals with type 2 diabetes mellitus (T2DM) increases. Elderly individuals with type 2 diabetes frequently experience various problems as a result of multiorgan aging, system function decline, and lowered immunity. The most prevalent consequence is a urinary tract infection (UTI). There are various factors that lead to urinary tract infection in older people with type 2 diabetes; however, there is a lack of systematic reviews and meta-analyses of these risk factors. As a result, this study used a systematic review method to identify the primary risk factors for UTI in T2D patients, providing a reference for preventing or reducing urinary tract infections in older people with type 2 diabetes.

19. * Participants/population.

Specify the participants or populations being studied in the review. The preferred format includes details of both inclusion and exclusion criteria.

Elderly patients with type 2 diabetes.

20. * Intervention(s), exposure(s).

Give full and clear descriptions or definitions of the interventions or the exposures to be reviewed. The preferred format includes details of both inclusion and exclusion criteria.

To explore the risk factors for urinary tract infection in elderly patients with type 2 diabetes mellitus.

21. * Comparator(s)/control.

Where relevant, give details of the alternatives against which the intervention/exposure will be compared (e.g. another intervention or a non-exposed control group). The preferred format includes details of both inclusion and exclusion criteria.

Non-exposed control group)

22. * Types of study to be included.

Give details of the study designs (e.g. RCT) that are eligible for inclusion in the review. The preferred format includes both inclusion and exclusion criteria. If there are no restrictions on the types of study, this should be stated.

Case-control studies and cohort studies

23. Context.

Give summary details of the setting or other relevant characteristics, which help define the inclusion or exclusion criteria.

24. * Main outcome(s).

Give the pre-specified main (most important) outcomes of the review, including details of how the outcome is defined and measured and when these measurement are made, if these are part of the review inclusion criteria.

Univariate and multivariate risk factors for UTIs in older persons with type 2 diabetes. Contains all physiological (general/medical), clinical, social, and demographic risk factors for UTI in older persons with type 2 diabetes.

Measures of effect

Please specify the effect measure(s) for you main outcome(s) e.g. relative risks, odds ratios, risk difference, and/or 'number needed to treat.

25. * Additional outcome(s).

List the pre-specified additional outcomes of the review, with a similar level of detail to that required for main outcomes. Where there are no additional outcomes please state ‘None’ or ‘Not applicable’ as appropriate to the review

Not applicable

Measures of effect

Please specify the effect measure(s) for you additional outcome(s) e.g. relative risks, odds ratios, risk difference, and/or 'number needed to treat.

26. * Data extraction (selection and coding).

Describe how studies will be selected for inclusion. State what data will be extracted or obtained. State how this will be done and recorded.

All data will be independently searched, screened, extracted, and cross-checked="checked" value="1" by two members of the research group.The two researchers independently performed study screening and data extraction. In the process of literature screening, the researcher first eliminated the duplicate literature, then carried out the initial screening by reading the literature title and abstract, and then re-screened by reading the full text to determine that the literature was finally included in the study. The third researcher evaluated the literature to see if there was disagreement, and the literature screening was carried out in strict accordance with the established inclusion and exclusion criteria. The two researchers independently extracted data from the included studies, and the data extraction contents were: author, year of publication, study design, study type, sample size, and various risk factors, etc. After the data is extracted, it will be summarized and exchanged for review, and if there is a disagreement, a third party will be invited to arbitrate.

27. * Risk of bias (quality) assessment.

State which characteristics of the studies will be assessed and/or any formal risk of bias/quality assessment tools that will be used.

The Newcastle-Ottawa Scale (NOS) is commonly used quality assessment tool for case-control studies.
and cohort studies. It evaluates [illegible] a total of

eight items. Specifically, it included study population selection, comparability, exposure, and outcome. NOS used the semi-quantization principle of the star system to evaluate the quality of literature. The full score is 9. and articles with less than 6 points are excluded. If two researchers disagree on the quality evaluation of the article, please ask a third researcher with the same qualification to decide.

28. * Strategy for data synthesis.

Describe the methods you plan to use to synthesise data. This **must not be generic text** but should be **specific to your review** and describe how the proposed approach will be applied to your data. If meta-analysis is planned, describe the models to be used, methods to explore statistical heterogeneity, and software package to be used.

The revman5.4 software was used for statistical analysis, and the stata17.0 software was used for the evaluation of sensitivity and publication bias. For continuous outcome data, the standard mean difference (SMD) or weighted mean difference (WMD) with a 95% CI (confidence interval) will be used. For dichotomous data, the risk ratio (RR) or risk difference (RD) with a 95% CI will be used for analysis.

29. * Analysis of subgroups or subsets.

State any planned investigation of ‘subgroups’. Be clear and specific about which type of study or participant will be included in each group or covariate investigated. State the planned analytic approach.

Subgroup analysis will be carried out when sufficient data is available. We will calculate subgroup analyses.
of gender, region, type of complications, etc.

30. * Type and method of review.

Select the type of review, review method and health area from the lists below.

Type of review

Cost effectiveness

No

Diagnostic

No

Epidemiologic

No

Individual patient data (IPD) meta-analysis

No

Intervention

No

Living systematic review

No

Meta-analysis

Yes

Methodology

No

Narrative synthesis

No

Network meta-analysis

No

Pre-clinical

No

Prevention

No

Prognostic

No

Prospective meta-analysis (PMA)

No

Review of reviews

No

Service delivery

No

Synthesis of qualitative studies

No

Systematic review
Yes

Other
No

Health area of the review

Alcohol/substance misuse/abuse
No

Blood and immune system
No

Cancer
No

Cardiovascular
No

Care of the elderly
No

Child health
No

Complementary therapies
No

COVID-19
No

Crime and justice
No

Dental
No

Digestive system
No

Ear, nose and throat
No

Education
No

Endocrine and metabolic disorders
No

Eye disorders

No

General interest

No

Genetics

No

Health inequalities/health equity

No

Infections and infestations

No

International development

No

Mental health and behavioural conditions

No

Musculoskeletal

No

Neurological

No

Nursing

Yes

Obstetrics and gynaecology

No

Oral health

No

Palliative care

No

Perioperative care

No

Physiotherapy

No

Pregnancy and childbirth

No

Public health (including social determinants of health)

No

Rehabilitation

No

Respiratory disorders

No

Service delivery

No

Skin disorders

No

Social care

No

Surgery

No

Tropical Medicine

No

Urological

No

Wounds, injuries and accidents

No

Violence and abuse

No

31. Language.

Select each language individually to add it to the list below, use the bin icon to remove any added in error.

English

There is not an English language summary

32. * Country.

Select the country in which the review is being carried out. For multi-national collaborations select all the countries involved.

China

33. Other registration details.

Name any other organisation where the systematic review title or protocol is registered (e.g. Campbell, or The Joanna Briggs Institute) together with any unique identification number assigned by them. If extracted data will be stored and made available through a repository such as the Systematic Review Data Repository (SRDR), details and a link should be included here. If none, leave blank.

34. Reference and/or URL for published protocol.

If the protocol for this review is published provide details (authors, title and journal details, preferably in Vancouver format)

Add web link to the published protocol.

Or, upload your published protocol here in pdf format. Note that the upload will be publicly accessible.

No I do not make this file publicly available until the review is complete

Please note that the information required in the PROSPERO registration form must be completed in full even if access to a protocol is given.

35. Dissemination plans.

Do you intend to publish the review on completion?

No

Give brief details of plans for communicating review findings.?

36. Keywords.

Give words or phrases that best describe the review. Separate keywords with a semicolon or new line. Keywords help PROSPERO users find your review (keywords do not appear in the public record but are included in searches). Be as specific and precise as possible. Avoid acronyms and abbreviations unless these are in wide use.

37. Details of any existing review of the same topic by the same authors.

If you are registering an update of an existing review give details of the earlier versions and include a full bibliographic reference, if available.

38. * Current review status.

Update review status when the review is completed and when it is published.New registrations must be ongoing so this field is not editable for initial submission.

Please provide anticipated publication date

Review_Ongoing

39. Any additional information.

Provide any other information relevant to the registration of this review.

40. Details of final report/publication(s) or preprints if available.

Leave empty until publication details are available OR you have a link to a preprint (NOTE: this field is not editable for initial submission). List authors, title and journal details preferably in Vancouver format.

Give the link to the published review or preprint.
