# Supplementary material for: Risk factors for urinary tract infection in elderly patients with type 2 diabetes: A protocol for systematic review and meta-analysis
Source: PLoS One. 2024 Sep 26;19(9):e0310903. doi: 10.1371/journal.pone.0310903 (PMC11426445; doi:10.1371/journal.pone.0310903)
Supplement: S3 File — (DOCX) [file pone.0310903.s004.docx]

**S3 File-Retrieval strategy**

**PubMed**

| Search number | Query | Results |
| --- | --- | --- |
| 1 | "Aged"[Mesh] | 3,515,493 |
| 2 | (Aged[Title/Abstract]) OR (Elderly[Title/Abstract]) | 1,022,187 |
| 3 | #1 OR #2 | 4,112,419 |
| 4 | "Diabetes Mellitus, Type 2"[Mesh] | 180,605 |
| 5 | (((((((((((((((((((((((((((((((Diabetes Mellitus, Type 2[Title/Abstract]) OR (Diabetes Mellitus, Adult-Onset[Title/Abstract])) OR (Adult-Onset Diabetes Mellitus[Title/Abstract])) OR (Diabetes Mellitus, Adult Onset[Title/Abstract])) OR (Diabetes Mellitus, Ketosis-Resistant[Title/Abstract])) OR (Diabetes Mellitus, Ketosis Resistant[Title/Abstract])) OR (Ketosis-Resistant Diabetes Mellitus[Title/Abstract])) OR (Diabetes Mellitus, Non Insulin Dependent[Title/Abstract])) OR (Diabetes Mellitus, Non-Insulin-Dependent[Title/Abstract])) OR (Non-Insulin-Dependent Diabetes Mellitus[Title/Abstract])) OR (Diabetes Mellitus, Stable[Title/Abstract])) OR (Stable Diabetes Mellitus[Title/Abstract])) OR (Diabetes Mellitus, Type II[Title/Abstract])) OR (NIDDM[Title/Abstract])) OR (Diabetes Mellitus, Noninsulin Dependent[Title/Abstract])) OR (Diabetes Mellitus, Maturity-Onset[Title/Abstract])) OR (Diabetes Mellitus, Maturity Onset[Title/Abstract])) OR (Maturity-Onset Diabetes Mellitus[Title/Abstract])) OR (Maturity Onset Diabetes Mellitus[Title/Abstract])) OR (MODY[Title/Abstract])) OR (Diabetes Mellitus, Slow-Onset[Title/Abstract])) OR (Diabetes Mellitus, Slow Onset[Title/Abstract])) OR (Slow-Onset Diabetes Mellitus[Title/Abstract])) OR (Type 2 Diabetes Mellitus[Title/Abstract])) OR (Noninsulin-Dependent Diabetes Mellitus[Title/Abstract])) OR (Noninsulin Dependent Diabetes Mellitus[Title/Abstract])) OR (Maturity-Onset Diabetes[Title/Abstract])) OR (Diabetes, Maturity-Onset[Title/Abstract])) OR (Maturity Onset Diabetes[Title/Abstract])) OR (Type 2 Diabetes[Title/Abstract])) OR (Diabetes, Type 2[Title/Abstract])) OR (Diabetes Mellitus, Noninsulin-Dependent[Title/Abstract]) | 195,073 |
| 6 | #4 OR #5 | 247,745 |
| 7 | "Urinary Tract Infections"[Mesh] | 51,787 |
| 8 | (((((Urinary Tract Infections[Title/Abstract]) OR (Infections, Urinary Tract[Title/Abstract])) OR (Infection, Urinary Tract[Title/Abstract])) OR (Tract Infections, Urinary[Title/Abstract])) OR (Tract Infection, Urinary[Title/Abstract])) OR (Urinary Tract Infection[Title/Abstract]) | 53,090 |
| 9 | #7 OR #8 | 77,369 |
| 10 | "Risk Factors"[Mesh] | 987,835 |
| 11 | ((((((((((((((((((Risk Factors[Title/Abstract]) OR (Factor, Risk[Title/Abstract])) OR (Risk Factor[Title/Abstract])) OR (Population at Risk[Title/Abstract])) OR (Populations at Risk[Title/Abstract])) OR (Risk Scores[Title/Abstract])) OR (Risk Score[Title/Abstract])) OR (Score, Risk[Title/Abstract])) OR (Risk Factor Scores[Title/Abstract])) OR (Risk Factor Score[Title/Abstract])) OR (Score, Risk Factor[Title/Abstract])) OR (Health Correlates[Title/Abstract])) OR (Correlates, Health[Title/Abstract])) OR (Social Risk Factors[Title/Abstract])) OR (Factor, Social Risk[Title/Abstract])) OR (Factors, Social Risk[Title/Abstract])) OR (Risk Factor, Social[Title/Abstract])) OR (Risk Factors, Social[Title/Abstract])) OR (Social Risk Factor[Title/Abstract]) | 860,625 |
| 12 | #10 OR #11 | 1,467,518 |
| 13 | #3 AND #6 AND #9 AND #12 | 85 |

**Embase**

No. Query Results

#1 'aged'/exp 3982096

#2 'aged':ab,ti 1060721

#3 'elderly':ab,ti 425295

#4 #1 OR #2 OR #3 4781312

#5 'non insulin dependent diabetes mellitus'/exp 360862

#6 'diabetes mellitus, type 2':ab,ti 6233

#7 'diabetes mellitus, adult-onset':ab,ti 3

#8 'diabetes mellitus, adult onset':ab,ti 3

#9 'diabetes mellitus, ketosis-resistant':ab,ti 0

#10 'diabetes mellitus, ketosis resistant':ab,ti 0

#11 'ketosis-resistant diabetes mellitus':ab,ti 2

#12 'diabetes mellitus, non insulin dependent':ab,ti 43

#13 'diabetes mellitus, non-insulin-dependent':ab,ti 43

#14 'non-insulin-dependent diabetes mellitus':ab,ti 7904

#15 'diabetes mellitus, stable':ab,ti 17

#16 'stable diabetes mellitus':ab,ti 27

#17 'non insulin dependent diabetes mellitus':ab,ti 7902

#18 'niddm':ab,ti 8133

#19 'diabetes mellitus, noninsulin dependent':ab,ti 5

#20 'diabetes mellitus, maturity-onset':ab,ti 15

#21 'diabetes mellitus, maturity onset':ab,ti 15

#22 'maturity-onset diabetes mellitus':ab,ti 174

#23 'maturity onset diabetes mellitus':ab,ti 174

#24 'mody':ab,ti 2983

#25 'diabetes mellitus, slow-onset':ab,ti 1

#26 'diabetes mellitus, slow onset':ab,ti 1

#27 'slow-onset diabetes mellitus':ab,ti 0

#28 'type 2 diabetes mellitus':ab,ti 93688

#29 'noninsulin-dependent diabetes mellitus':ab,ti 1067

#30 'noninsulin dependent diabetes mellitus':ab,ti 1067

#31 'maturity-onset diabetes':ab,ti 3196

#32 'diabetes, maturity-onset':ab,ti 59

#33 'maturity onset diabetes':ab,ti 3196

#34 'type 2 diabetes':ab,ti 266913

#35 'diabetes, type 2':ab,ti 2994

#36 'diabetes mellitus, noninsulin-dependent':ab,ti 5

#37 #5 OR #6 OR #7 OR #8 OR #9 OR #10 OR #11 OR #12 OR #13 OR #14 OR #15 OR #16 OR #17 OR #18 OR #19 OR #20 OR #21 OR #22 OR #23 OR #24 OR #25 OR #26 OR #27 OR #28 OR #29 OR #30 OR #31 OR #32 OR #33 OR #34 OR #35 OR #36 410166

#38 'urinary tract infection'/exp 158432

#39 'urinary tract infection':ab,ti 45399

#40 'infections, urinary tract':ab,ti 499

#41 'infection, urinary tract':ab,ti 545

#42 'tract infections, urinary':ab,ti 181

#43 'tract infection, urinary':ab,ti 198

#44 'urinary tract infection':ab,ti 45399

#45 #38 OR #39 OR #40 OR #41 OR #43 OR #44 164855

#46 'risk factor'/exp 1432536

#47 'risk factor':ab,ti 414965

#48 'factor, risk':ab,ti 352

#49 'risk factor':ab,ti 414965

#50 'population at risk':ab,ti 6060

#51 'populations at risk':ab,ti 3751

#52 'risk score':ab,ti 50731

#53 'risk scores':ab,ti 23902

#54 'score, risk':ab,ti 1361

#55 'risk factor scores':ab,ti 150

#56 'risk factor score':ab,ti 301

#57 'score, risk factor':ab,ti 17

#58 'health correlates':ab,ti 635

#59 'correlates, health':ab,ti 12

#60 'social risk factors':ab,ti 1456

#61 'factor, social risk':ab,ti 0

#62 'factors, social risk':ab,ti 9

#63 'risk factor, social':ab,ti 7

#64 'risk factors, social':ab,ti 150

#65 'social risk factor':ab,ti 120

#66 #46 OR #47 OR #48 OR #49 OR #50 OR #51 OR #52 OR #53 OR #54 OR #55 OR #56 OR #57 OR #58 OR #59 OR #60 OR #61 OR #62 OR #63 OR #64 OR #65 1652466

#67 #4 AND #37 AND #45 AND #66 184

**Cochrane library**

ID Search Hits

#1 MeSH descriptor: [Aged] explode all trees 275455

#2 (Aged):ti,ab,kw OR (Elderly):ti,ab,kw 678765

#3 #1 or #2 678765

#4 MeSH descriptor: [Diabetes Mellitus, Type 2] explode all trees 26311

#5 (Diabetes Mellitus, Type 2):ti,ab,kw OR (Diabetes Mellitus, Adult-Onset):ti,ab,kw OR (Adult-Onset Diabetes Mellitus):ti,ab,kw OR (Diabetes Mellitus, Adult Onset):ti,ab,kw OR (Diabetes Mellitus, Ketosis-Resistant):ti,ab,kw 55126

#6 (Diabetes Mellitus, Ketosis Resistant):ti,ab,kw OR (Ketosis-Resistant Diabetes Mellitus):ti,ab,kw OR (Diabetes Mellitus, Non Insulin Dependent):ti,ab,kw OR (Diabetes Mellitus, Non-Insulin-Dependent):ti,ab,kw OR (Non-Insulin-Dependent Diabetes Mellitus):ti,ab,kw 23716

#7 (Diabetes Mellitus, Stable):ti,ab,kw OR (Stable Diabetes Mellitus):ti,ab,kw OR (Diabetes Mellitus, Type II):ti,ab,kw OR (NIDDM):ti,ab,kw OR (Diabetes Mellitus, Noninsulin Dependent):ti,ab,kw 10520

#8 (Diabetes Mellitus, Maturity-Onset):ti,ab,kw OR (Diabetes Mellitus, Maturity Onset):ti,ab,kw OR (Maturity-Onset Diabetes Mellitus):ti,ab,kw OR (Maturity Onset Diabetes Mellitus):ti,ab,kw OR (MODY):ti,ab,kw 111

#9 (Diabetes Mellitus, Slow-Onset):ti,ab,kw OR (Diabetes Mellitus, Slow Onset):ti,ab,kw OR (Slow-Onset Diabetes Mellitus):ti,ab,kw OR (Type 2 Diabetes Mellitus):ti,ab,kw OR (Noninsulin-Dependent Diabetes Mellitus):ti,ab,kw 54571

#10 (Noninsulin Dependent Diabetes Mellitus):ti,ab,kw OR (Maturity-Onset Diabetes):ti,ab,kw OR (Diabetes, Maturity-Onset):ti,ab,kw OR (Maturity Onset Diabetes):ti,ab,kw OR (Type 2 Diabetes):ti,ab,kw 62289

#11 (Diabetes, Type 2):ti,ab,kw OR (Diabetes Mellitus, Noninsulin-Dependent):ti,ab,kw 62218

#12 #4 or #5 or #6 or #7 or #8 or #9 or #10 or #11 66474

#13 MeSH descriptor: [Urinary Tract Infections] explode all trees 3305

#14 (Urinary Tract Infections):ti,ab,kw OR (Infection, Urinary Tract):ti,ab,kw OR (Infection, Urinary Tract):ti,ab,kw OR (Tract Infections, Urinary):ti,ab,kw OR (Tract Infection, Urinary):ti,ab,kw 11063

#15 (Urinary Tract Infection):ti,ab,kw 9331

#16 #13 or #14 or #15 11398

#17 MeSH descriptor: [Risk Factors] explode all trees 38174

#18 (Risk Factors):ti,ab,kw OR (Factor, Risk):ti,ab,kw OR (Risk Factor):ti,ab,kw OR (Population at Risk):ti,ab,kw OR (Populations at Risk):ti,ab,kw 156588

#19 (Risk Scores):ti,ab,kw OR (Risk Score):ti,ab,kw OR (Score, Risk):ti,ab,kw OR (Risk Factor Scores):ti,ab,kw OR (Risk Factor Score):ti,ab,kw 53976

#20 (Score, Risk Factor):ti,ab,kw OR (Health Correlates):ti,ab,kw OR (Correlates, Health):ti,ab,kw OR (Social Risk Factors):ti,ab,kw OR (Factor, Social Risk):ti,ab,kw 15963

#21 (Factors, Social Risk):ti,ab,kw OR (Risk Factor, Social):ti,ab,kw OR (Risk Factors, Social):ti,ab,kw OR (Social Risk Factor):ti,ab,kw 7183

#22 #17 or #18 or #19 or #20 or #21 184065

#23 #3 and #12 and #16 and #22 91

**Web of science**

# Search Query Results

1 "TS=(Aged) OR TS=( Elderly) 2549195

2 "TS=(Diabetes Mellitus, Type 2) OR TS=(Diabetes Mellitus, Adult-Onset) OR TS=(Adult-Onset Diabetes Mellitus) OR TS=(Diabetes Mellitus, Adult Onset) OR TS=(Diabetes Mellitus, Ketosis-Resistant) OR TS=(Diabetes Mellitus, Ketosis Resistant) OR TS=(Ketosis-Resistant Diabetes Mellitus) OR TS=(Diabetes Mellitus, Non Insulin Dependent) OR TS=(Diabetes Mellitus, Non-Insulin-Dependent) OR TS=(Non-Insulin-Dependent Diabetes Mellitus)

OR TS=(Diabetes Mellitus, Stable) OR TS=(Stable Diabetes Mellitus) OR TS=(Diabetes Mellitus, Type II) OR TS=(NIDDM) OR TS=(Diabetes Mellitus, Noninsulin Dependent) OR TS=(Diabetes Mellitus, Maturity-Onset) OR TS=(Diabetes Mellitus, Maturity Onset) OR TS=(Maturity-Onset Diabetes Mellitus) OR TS=(Maturity Onset Diabetes Mellitus) OR TS=(MODY) OR TS=(Diabetes Mellitus, Slow-Onset) OR TS=(Diabetes Mellitus, Slow Onset) OR TS=(Slow-Onset Diabetes Mellitus) OR TS=(Type 2 Diabetes Mellitus) OR TS=(Noninsulin-Dependent Diabetes Mellitus) OR TS=(Noninsulin Dependent Diabetes Mellitus) OR TS=(Maturity-Onset Diabetes) OR TS=(Diabetes, Maturity-Onset) OR TS=(Maturity Onset Diabetes) OR TS=(Type 2 Diabetes) OR TS=(Diabetes, Type 2) OR TS=(Diabetes Mellitus, Noninsulin-Dependent) 215201

3 "TS=(Urinary Tract Infections) OR TS=(Infections, Urinary Tract) OR TS=(Infection, Urinary Tract) OR TS=(Tract Infections, Urinary) OR TS=(Tract Infection, Urinary) OR TS=(Urinary Tract Infection) 34577

4 "TS=(Risk Factors) OR TS=(Factor, Risk) OR TS=(Risk Factor) OR TS=(Population at Risk) OR TS=(Populations at Risk) OR TS=(Risk Scores) OR TS=(Risk Score) OR TS=(Score, Risk) OR TS=(Risk Factor Scores) OR TS=(Risk Factor Score) OR TS=(Score, Risk Factor) OR TS=(Health Correlates) OR TS=(Correlates, Health) OR TS=(Social Risk Factors) OR TS=(Factor, Social Risk) OR TS=(Factors, Social Risk) OR TS=(Risk Factor, Social) OR TS=(Risk Factors, Social) OR TS=(Social Risk Factor) 1526193

5 "#1 AND #2 AND #3 AND #4 134

**WanFang**

1 题目或关键词（老人 OR 老年人 OR 老年）808883

2 题目或关键词 (2型糖尿病 OR 成人发病型糖尿病 OR 2型糖尿病 OR 非胰岛素依赖性糖尿病 OR 非胰岛素依赖型糖尿病 OR 二型糖尿病） 122945

3 题目或关键词 (尿路感染 OR 泌尿系统感染 OR 尿感 OR 尿道感染 OR 泌尿系感染 OR 泌尿道感染 OR 泌尿感染) 54858

4 题目或关键词 (危险因素 OR 影响因素 OR 预测因素) 729435

5 1 and 2 and 3 and 4 38
